# The complete chloroplast genome sequence of *Ficus concinna* (Moraceae) from Sichuan province

**DOI:** 10.1080/23802359.2022.2036649

**Published:** 2022-02-20

**Authors:** Pei-hua Zhang, Zhu Hou

**Affiliations:** a Zhang Zhongjing College of Chinese Medicine, Nanyang institute of Technology, Nanyang, China; b China West Normal University, Nanchong, Sichuan, China

**Keywords:** *Ficus concinna*, chloroplast genome, phylogenetic analysis

## Abstract

*Ficus concinna* is an important contributor to tropical forest biodiversity. Here, we provide the first report of the complete *F. concinna* chloroplast genome, thereby providing a basis for subsequent phylogenetic analyses of the *Moraceae* family. The final assembled chloroplast genome was 160,331 bp in size and included a 20,018 bp long small single-copy (SSC) region, an 88,541 bp long large single-copy (LSC) region, and two 25,886 bp inverted repeats (IRs). The total content of GC for this chloroplast genome was 42.6%, with respective frequencies of 33.6%, 35.9%, and 28.9% in the SSC, LSC, and IR regions, accordingly. Overall, the chloroplast genome was found to encode 131 genes, comprising 37 tRNAs, 86 protein-coding genes, and 8 rRNAs. Phylogenetic analyses revealed a close relationship between *F. concinna* and *Ficus altissima*, consistent with prior research results. Together, these data provide valuable insights regarding the evolution and conserved genetic features of *F. concinna*.

*Ficus concinna* Miq. (Ann. 1867) is an important contributor to tropical forest biodiversity (Davis et al. 2002). The *Ficus* genus includes an estimated 735 species, and serves as a valuable subject for studies of symbiotic relationships between pollinators and flowers (Berg and Corner 2005). This genus includes figs, which are important members of several ecosystems in addition to functioning as an important culinary and medicinal resource. *Ficus* L. species are the most widely distributed genera in tropical regions where they serve as keystone species given the complex obligatory mutualistic relationship between the figs and the agaonid fig wasps which pollinate these plants (Dunn 2020).

*Ficus concinna* is among the oldest known cultivated food sources, yet it remains highlight susceptible to soil-transmitted Ceratocystis canker and other fungal diseases (Kajitani and Masuya 2011). *F. concinna* is a monoecious species of fig tree that is found in many regions of Asia (Berg and Corner 2005), and it is endemic to tropical forests in Xishuangbanna, Yunnan, China. Furthermore, *F. concinna* is commonly planted as an ornamental or sacred tree near temples or in villages. The branches and aerial roots of this species are employed in traditional Chinese medicine, while its tender leaves serve as an edible vegetable for the residents of the Xishuangbanna region. High-throughput sequencing-based analyses of the phylogenetic relationships between *F. concinna* and other *Ficus* species are necessary to provide a more robust foundation for comparisons among these species. Here, we utilized an Illumina paired-end sequencing approach to fully sequence the *F. concinna* chloroplast genome and to subsequently compare these sequences with previously published chloroplast genomes from other members of the *Ficus* genus. These results offer valuable insight into the evolution and conserved genetic features of *F. concinna*.

The samples of *F. concinna* were obtained from Nanchong, Sichuan Province, China (106°08′E; 30°79′N), with a sample having been deposited at the NanYang Medical College Herbarium, Department of Chinese Medicine (https://www.nyist.edu.cn/, Pei-hua Zhang, guoguof1010@163.com; voucher number XYR001). The extraction of total genomic DNA (gDNA) was executed from healthy, fresh leaves with a DNA Secure Plant Kit (Tiangen Biotech, Beijing, China) based on provided directions and stored at −80 °C. A Mosquito LV (SPT Labtech) was used with a Nextera XT DNA library preparation kit (Illumina) for the preparation of DNA libraries, with DNA template samples being barcoded prior to pooling in single lane for paired-end Illumina MiSeq v2 300 bp sequencing on the Illumina HiSeq 4000 platform (Illumina, CA, USA). This analysis yielded 5.4 Gb of raw reads in total, with raw sequencing data having been deposited in the NCBI SRA database (accession: SRR14793492). Trimmomatic v 0.32 was used to filter raw data using predefined settings (Bolger et al. 2014). Left cleaned reads were assembled into a chloroplast genome employing SPAdes v.3.9.0 (Nurk et al. 2017). The MPI-MP CHLOROBOX (https://chlorobox.mpimp-golm.mpg.de/geseq.html) was implemented for analyzing the annotated sequence data with GeSeq (Tillich et al. 2017). The published *Ficus altissima* chloroplast genome (NC053895) served as a reference for these analyses, with Geneious Prime v2020.2 being (Kearse et al. 2012). The final *F. concinna* chloroplast genome sequence was then submitted to GenBank (Accession No. MZ128521).

The assembled chloroplast genome was 160,331 bp long and composed of a 20,018 bp small single-copy (SSC) region, an 88,541 bp large single-copy (LSC) region, and two 25,886 bp inverted repeat (IR) regions. The total content of GC for this chloroplast genome was 42.6%, with respective frequencies of 33.6%, 35.9%, and 28.9% in the SSC, LSC, and IR regions, accordingly. Overall, the chloroplast genome was found to encode 131 genes, comprising 37 tRNAs, 86 protein-coding genes, and 8 rRNAs. The *F. concinna* plastome was 14 bp in length, making it 10 bp longer than that of *F. altissima* (NC053895) and *F. microcarpa* (152,719 bp, NC053834), whereas it was 31 and 46 bp smaller, respectively, compared to those of *F. curtipes* (152,760 bp, NC053833) and *F. religlosa* (152,775 bp, NC033979). *C. septentrionale* exhibited an overall GC content of 39.1%, in line with similar results for *F. altissima* (NC053895), *F. microcarpa* (NC053834), and *F. religlosa* (NC033979).

To more fully clarify the taxonomic relationships between *F. concinna* and similar species, MEGA 7.0 was next employed to construct a maximum likelihood (ML) phylogenetic tree based upon the complete chloroplast genome sequence data generated above (Kumar et al. 2016). The parameters used to construct this tree included: a Tamura 3-parameter (T92) nucleotide substitution model with 1000 bootstrap replicates, missing data or partial deletion for gaps, and Gamma distributed with invariant sites (G + I). The complete chloroplast genome sequences for an outgroup species (*Artocarpus hypargyreus*) and 16 additional *Ficus* species were obtained from the GenBank database. MAFFT was then used to align these 18 chloroplast genome sequences (Katoh and Standley 2013), with the ML tree being used to establish phylogenetic relationships between *F. concinna* and these other species (Figure 1). All nodes within the resultant tree exhibited strong bootstrap values (>50%). *F. concinna* was located in the same clade as *F. altissima*, *F. microcarpa*, *F. curtipes,* and *F. religlosa*, forming a fully supported *Ficus* sister clade, with *F. altissima* being the most closely related of these species. *F. concinna* is an economically and ecologically important species found within subtropical and tropical forests. As the individual species within the *Ficus* genus do not exhibit definitive morphological characteristics that differentiate them from one another, this genera is well suited for DNA barcoding-based analyses aimed at differentiating among species and genera. The phylogenetic findings within the present report are consistent with prior barcode sequencing conclusions, with an rbcL + matK + trnH–psbA + ITS barcode-based analysis having revealed a close relationship between *F. concinna* and *F. altissima*. These data thus offer valuable insight regarding the evolution and genetic conservation of *F. concinna*.

**Figure 1.**
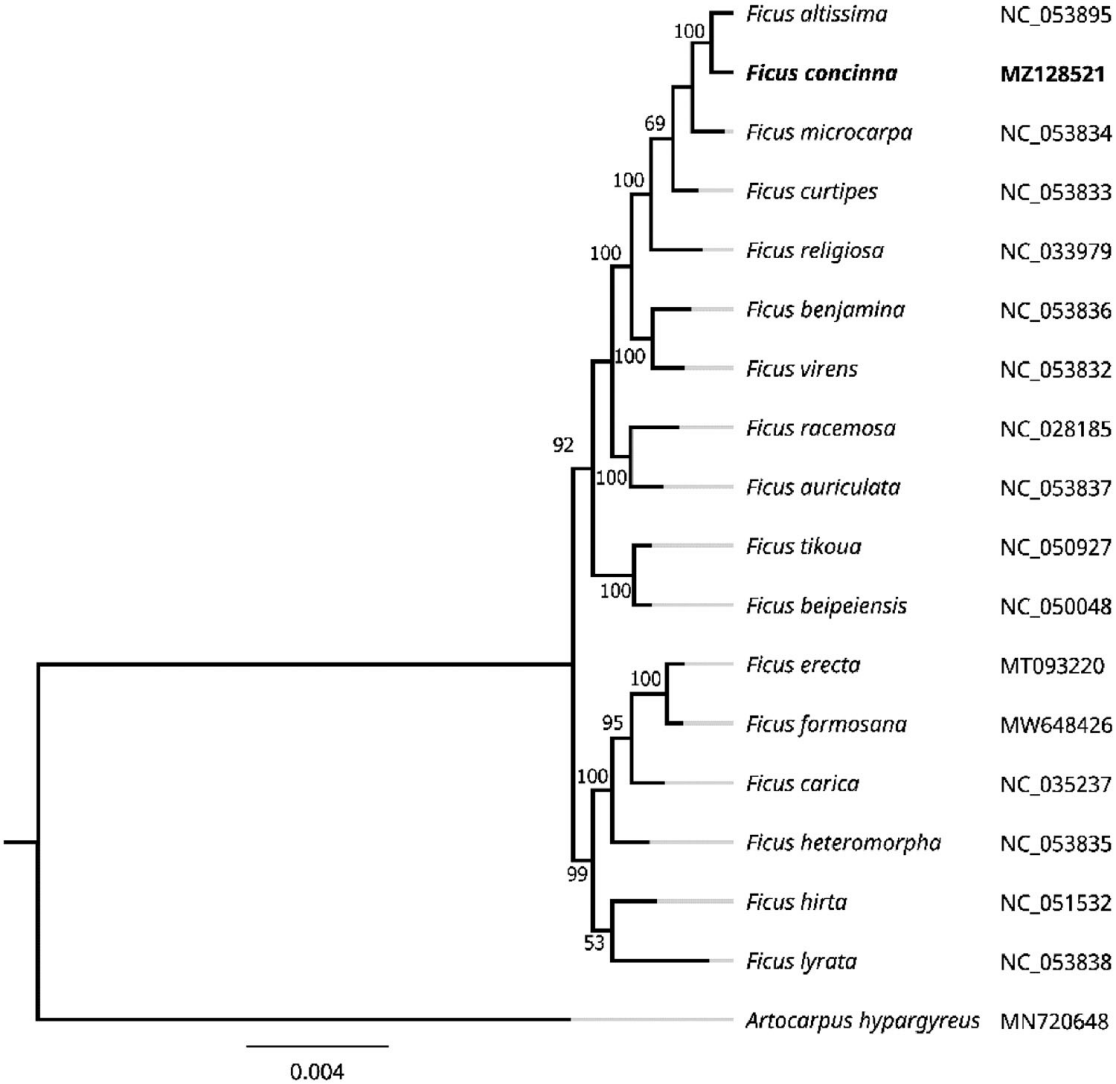
A chloroplast genome sequence-based maximum likelihood phylogenetic tree for *F. concinna* and related species.

## Data Availability

The genome sequence data that support the findings of this study are openly available in GenBank of NCBI at (https://www.ncbi.nlm.nih.gov/) under the accession No. MZ128521. The associated BioProject, SRA, and Bio-Sample numbers are PRJNA737038, SRR14793492, and SAMN19678359 respectively.
